# Adherence to severe malaria treatment guidelines in children at a Ugandan regional hospital: a baseline assessment for a malaria treatment quality improvement project

**DOI:** 10.1186/s12936-023-04507-4

**Published:** 2023-02-25

**Authors:** Cynthia A. Moffitt, Peter Olupot-Olupot, Joan Wamulugwa Onen, Nicole O’Brien

**Affiliations:** 1 Division of Pediatric Critical Care, Nationwide Children’s Hospital, 700 Children’s Drive, Columbus, OH 43205 USA; 2 Faculty of Health Sciences, Busitema University, Mbale Regional Referral Hospital, Mbale, Uganda; 3 Mbale Regional Referral Hospital, Mbale, Uganda

**Keywords:** Malaria, Severe malaria, Guidelines, Case management, Adherence, Uganda

## Abstract

**Background:**

Malaria is one of the most common causes of hospital admission and death in children under the age of five. The World Health Organization (WHO) has issued guidelines for the identification and treatment of severe malaria. Evidence has shown that adherence to standardized malaria treatment protocols improves outcomes. As a baseline assessment in preparation for a malaria treatment quality improvement project, this study aimed to determine adherence to the WHO severe malaria treatment guidelines in children at a Ugandan Regional Referral Hospital.

**Methods:**

A retrospective review was performed on a convenience sample of children discharged between June 2021 and March 2022 from the Mbale Regional Referral Hospital Paediatrics Ward with a diagnosis of severe malaria. Data were collected using a standardized case report form. Demographics, presenting symptoms, laboratory results, treatments, length of stay, and mortality were extracted. Comparison of treatments received to items recommended in the WHO guidelines was undertaken to determine adherence.

**Results:**

147 patients were included. The median age was 5 years (IQR 2–7 years), and 55% were male. The most common features of severe malaria were haemoglobinuria (49%), haemoglobin < 5 mg/dL (34%), and altered mentation (24%). Median hospital length of stay was 3 days (IQR 2–4 days), and the mortality rate was 27% (n = 40). Overall adherence to all aspects of the WHO severe malaria guidelines was achieved in 3% (n = 4) of patients. The most common areas of deficiency were not testing to confirm malaria diagnosis (34%) and inadequate administration of artesunate (82%). Fewer than the three recommended doses of artesunate occurred in 22% of patients. Additionally, a delay in the administration of the second dose occurred in 67% (n = 78) and in the third dose in 77% (n = 71) of patients. While the recommended time between doses is 12 h, the median interval between dose one and dose two was 15 h (12–20) and the median interval from dose two to dose three was 17 h (14–25).

**Conclusions:**

Current adherence to severe malaria treatment guidelines in children at this Ugandan regional referral hospital is poor, but this study has identified target areas for improvement.

## Background

Malaria remains one of the most significant burdens on the health of children throughout the world. According to the 2021 World Health Organization (WHO) World Malaria Report, there were 241 million cases of malaria in 2020 in 85 malaria-endemic countries. These cases led to 627,000 deaths, 77% of which were in children under 5 years of age. Uganda accounted for 5% of all malaria cases, making it the 3^rd^ most burdened country after Nigeria and the Democratic Republic of the Congo [1]. In Uganda, malaria remains the most common cause of outpatient health visits (29.1%), hospital admissions (39.5%), and the most common cause of death in health facilities (10.9%). This is particularly true for children under the age of 5, with 16.4% of deaths in health facilities being attributable to malaria—the leading cause of death in this age group [2].

In order to address these issues, the WHO has issued guidelines for the identification and treatment of malaria [3, 4]. These guidelines are mirrored by the Ugandan national guidelines issued by the Ministry of Health [5]. Unfortunately, studies have shown that adherence to these guidelines by clinicians in sub-Saharan Africa is generally poor, around 50% at best [6–8]. Encouragingly, a recent study from Sierra Leone identified avenues to improve adherence to a standardized malaria treatment protocol with subsequent reduced mortality in children admitted to the hospital with severe malaria [9]. The goals of this study were to assess the current adherence to guidelines for severe malaria in children at a Ugandan regional hospital and identify areas for intervention to improve this adherence as part of a baseline assessment for a malaria treatment quality improvement initiative.

## Methods

This is a retrospective descriptive study of children with severe malaria based on a convenience sample of children discharged between June 2021 and March 2022 from the Mbale Regional Referral Hospital Paediatrics Ward. This study was approved by the institutional review board at Nationwide Children’s Hospital, USA and the Mbale Regional Referral Hospital Research Ethics Committee, Mbale, Uganda.

The setting of this study is a government-funded regional referral hospital. As a public hospital, patients do not need to pay for care received at this hospital, nor do they pay for any medications, supplies, or laboratory tests that are available at the hospital. Similar to other regional and district hospitals in sub-Saharan Africa, supplies available at the hospital are limited and subject to frequent stock-outs. Long-term employees report that quarterly deliveries of supplies typically last 1–2 months. The paediatrics ward at this hospital serves 100–200 children each day on average. The ward is staffed by clinical officers, intern physicians, resident and attending paediatric physicians, qualified nurses, as well as medical students and nursing students. Physicians are present on the acute section of the ward from approximately 7:00am to 11:00 pm and otherwise available to be called in if necessary. From 11:00 pm to 7:00am there is usually one qualified nurse staffing the acute section of the ward. The providers rotate frequently, usually on a weekly basis. The acute section of the ward has two cardiorespiratory monitors and oxygen tanks available. The ward is equipped with a glucometer and malaria rapid diagnostic tests (RDTs), though glucometer strips and RDT materials frequently suffer from stock-outs. The hospital lab is able to perform complete blood counts, microscopy for malaria parasites, complete metabolic panels, urine and stool microscopy, and urinalyses. This laboratory is open during daytime hours on weekdays, and limited weekend hours. The most commonly prescribed medications and fluids used for patients described in this study are provided by the hospital when in stock. Similar to other hospitals in sub-Saharan Africa, a patient caregiver must be present to assist in care for the patient. The ward staff provide all intravenous medications and appropriate monitoring, but any oral medications, nutritional needs, and hygiene needs are the responsibility of the caregivers accompanying the patient.

The sample was selected on review of paper charts of children who had been discharged from the paediatrics ward. All paper charts of paediatric patients are stored within the ward after patient discharge for several months before entering long-term storage. Discharged patient’s charts available on the ward at the time of initiation of the study were assessed, and those with documentation of a diagnosis of severe malaria were further reviewed and data abstracted. Initial screening identified 172 children that had a clinician reported diagnosis of “severe malaria,” but on further review of diagnostic criteria, only 147 of these children had documented evidence of severe malaria following WHO criteria (Table 1) [3–5]. Those that were excluded did not have documentation of any of the criteria used by the WHO to define severe malaria in children.

**Table 1:** Summary of WHO Defining Criteria for Severe Malaria

Confirmed Malaria Parasitemia + Any of the Following
Altered mental status
Prostration
More than two seizures in 24 h
Metabolic acidosis with a base deficit of > 8 mEq/L or plasma bicarbonate < 15 mmol/L
Plasma lactate ≥ 5 mmol/L
Respiratory distress
Blood or plasma glucose < 40 mg/dL
Haemoglobin ≤ 5 g/dL or haematocrit ≤ 15%,
Haemoglobinuria
Serum creatinine > 3 mg/dL or blood urea > 20 mmol/L
Plasma or serum bilirubin > 3 mg/dL
Clinical or radiographic evidence of pulmonary edema
Significant abnormal bleeding
Capillary refill ≥ 3 s or systolic blood pressure < 50 mmHg

There were no additional exclusion criteria. Data were collected via manual chart review using a standardized case report form. Demographics, symptoms, laboratory results, treatments, length of stay, and mortality were extracted and entered in Microsoft excel for statistical analysis. Comparison of treatments received to standards as recommended in the WHO and Uganda Ministry of Health guidelines (Table 2) was undertaken [3–5].

**Table 2:** Summary of WHO and Uganda Ministry of Health Guidelines for Management of Severe Malaria in Children

**Recommendations for All Patients with Severe Malaria**
Laboratory Evaluation
Thick and thin blood films or rapid diagnostic test to confirm diagnosis of malaria
Haemoglobin or haematocrit
Blood glucose level
Blood culture if feasible
General Management
Admit patients to highest level of care available
Minimum 3 doses IV artesunate at admission, 12 h, and 24 h 3 mg/kg for children < 20 kg, 2.4 mg/kg for children ≥ 20 kg Following the parenteral course, patients should complete a full 3-day oral antimalarial course
Empiric treatment for likely bacterial co-infection with broad-spectrum antibiotics If bacterial co-infection cannot be ruled out by blood culture/CSF analysis, complete an empiric course
Correct dehydration and monitor urine output
Administer paracetamol to maintain core temperature < 39C
**Recommendations for Patients with Altered Mental Status**
Provide oxygen
Assess for and correct hypoglycaemia if present with 5 ml/kg of 10% dextrose
Insert nasogastric tube to prevent aspiration
Position in lateral or semi-prone position and turn every 2 h If concerns for increased intracranial pressure, position supine with head raised about 30°
Perform analysis of CSF
**Recommendations for Patients with Seizure Activity**
Provide Oxygen
Assess for and correct hypoglycaemia if present with 5 ml/kg of 10% dextrose
Treat seizures with IV or rectal diazepam up to 2 doses 10 min apart If seizures persist, load with phenytoin or phenobarbital
**Recommendations for Patients with Severe Anaemia**
Provide Oxygen
Give blood transfusions to correct severe anaemia 10 ml/kg of packed red blood cells or 20 ml/kg of whole blood
**Recommendations for Patients with Poor Perfusion**
Provide Oxygen
Assess for anaemia and give blood transfusion if appropriate 10 ml/kg of packed red blood cells or 20 ml/kg of whole blood
Assess for and correct dehydration and monitor urine output

The primary outcome for the study was adherence to treatment guidelines. Adherence to guidelines was assessed for each patient both as a whole (Complete Adherence) and for each guideline individually as it applied to that patient. Adherence to diagnostic testing guidelines was considered appropriate if the patient had a documented positive test for malaria before receiving their second dose of artesunate in order to account for emergency situations where the first dose was given at the time of presentation before a test result was available. A patient could have either a positive malaria rapid diagnostic test (RDT) or positive microscopy to be considered adherent. Adherence to artesunate administration guidelines was considered appropriate if the patient received the 3 minimum doses recommended and the doses were given at the recommended interval of 12 h. Based on local guidelines that recommend medications be provided within 1 h of ordered time, administration outside of this 1-h time window (greater than 13 h) was considered “delayed”. Adherence to artesunate administration guidelines in patients who died before the 3 minimum recommended doses were received was assessed only on those doses that should have been received prior to death. Adherence to haemoglobin monitoring guidelines was considered appropriate if the patient had a documented haemoglobin result, as this is recommended for all patients with severe malaria. Adherence to blood transfusion administration guidelines was considered appropriate if patients who had a haemoglobin < 5 mg/dL received a transfusion of packed red blood cells 10 ml/kg or whole blood 20 ml/kg, or if children who did not have a haemoglobin result were deemed clinically to require blood transfusion received the appropriate dose of products. Adherence to hypoglycaemia management guidelines was considered appropriate if patients either had a documented blood glucose result or received empiric treatment with 5 ml/kg of 10% dextrose fluids when presenting with altered mental status or seizure activity when a blood glucose result was not available.

Adherence to oxygen administration guidelines was considered appropriate if patients with respiratory distress, severe anaemia, altered mental status, seizure activity, or documented poor perfusion were placed on oxygen therapy. Adherence to seizure management guidelines was considered appropriate if patients with documented seizure activity received appropriate doses of diazepam or phenobarbital. Adherence to guidelines on nasogastric tube use in unconscious children was considered appropriate if patients noted to have altered mental status had a nasogastric tube placed. Adherence to guidelines concerning empiric antibiotic use was considered appropriate if patients received an empiric course of ceftriaxone. Complete adherence to all guidelines was defined as having all of the above relevant criteria met for each patient. Continuous data are reported as median (IQR). Categorical data are reported as percentages.

## Results

**Fig.1:**
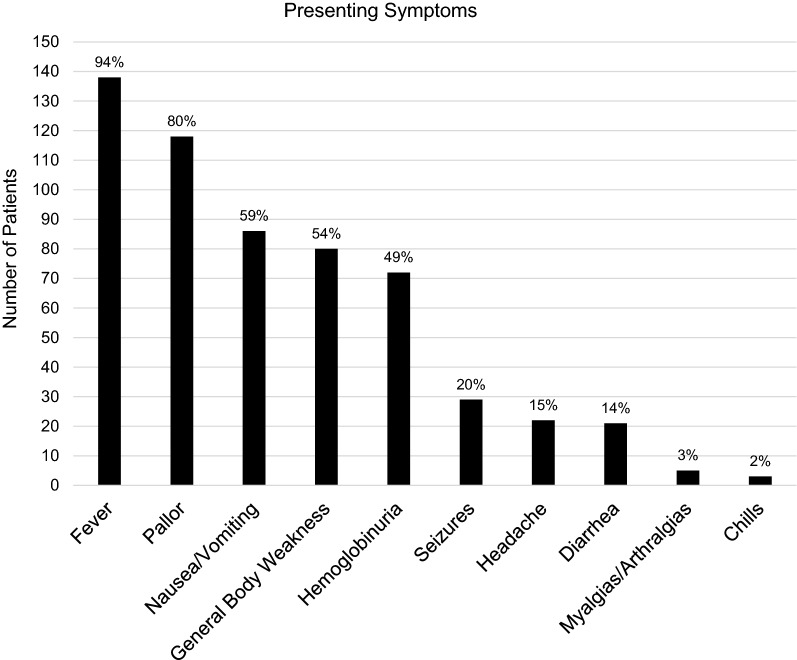
Most common presenting symptoms in children admitted with severe malaria (N = 147)

Among 147 patients who were included, 55% were male and the median age was 5 years (IQR 2–7 years). Table 3 shows overall patient characteristics. Hospital length of stay was 3 days (IQR 2–4 days), and the mortality rate in this sample was 27% (n = 40). Presenting symptoms of included patients are outlined in Fig. 1, with the most common complaint being fever in 94% of patients.

**Table 3:** Characteristics of the study population (N = 147)

Patient variable	Number of patients (%)
Age	
< 1 year	13 (9%)
1 year–5 years	74 (50%)
6 – 14 years	59 (40%)
Unknown	1 (1%)
Sex	
Male	81 (55%)
Female	56 (38%)
Unknown	10 (7%)
Discharge status	
Home	107 (73%)
Deceased	40 (27%)

**Fig. 2:**
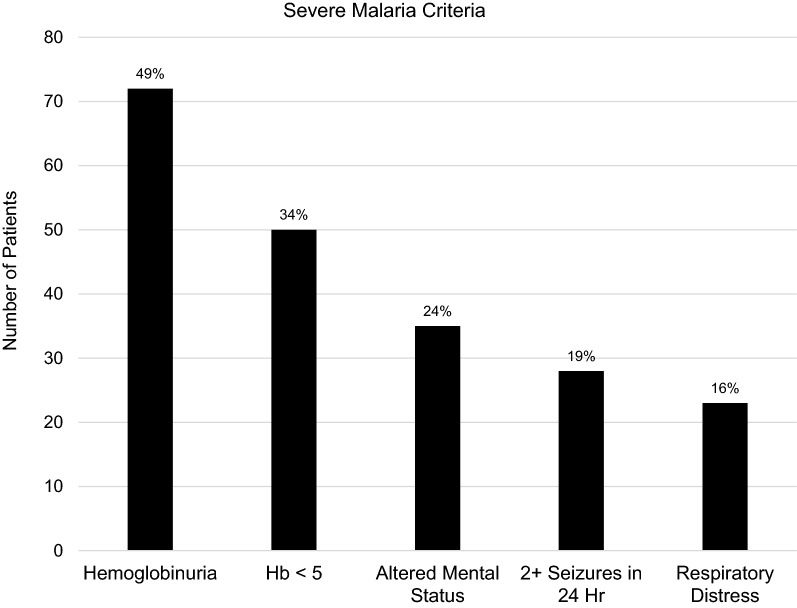
Most common features of severe malaria in the study population (N = 147). All criteria for severe malaria that were regularly measured or captured in the data are included in the figure

The most common features of severe malaria were haemoglobinuria (49%), haemoglobin < 5 mg/dL (34%), and altered mentation (24%) (Fig. 2).

Many other potential determinants of severe malaria were not regularly measured or captured, including metabolic acidosis, plasma lactate, blood glucose, markers of kidney injury, plasma or serum bilirubin, and blood pressures. Only 53% of patients had a haemoglobin documented. Among patients who had a documented pre-transfusion haemoglobin, 65% were less than 5 mg/dL.

**Fig. 3:**
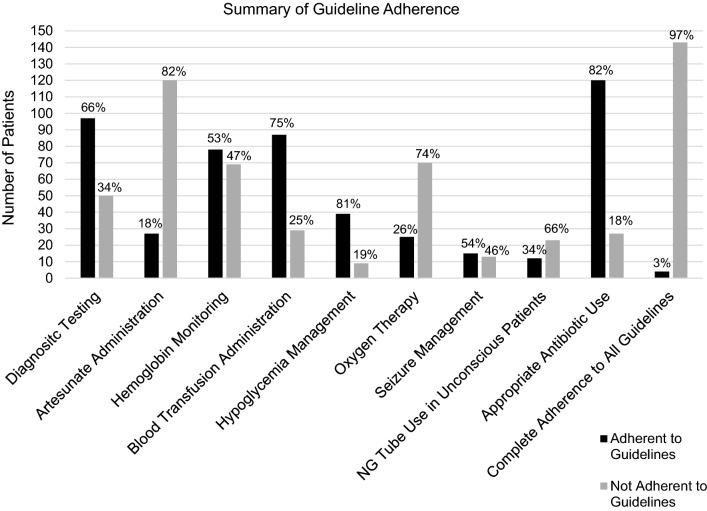
Summary of adherence to treatment guidelines in children with severe malaria. Each individual guideline adherence is presented for the subgroup of patients for which it was applicable as well as the complete adherence to all guidelines for all patients. The number of patients included in each individual guideline assessment are as follows: Diagnostic Testing N = 147, Artesunate Administration N = 147, Haemoglobin Monitoring N = 147, Blood Transfusion Administration N = 116, Hypoglycaemia Management N = 48, Oxygen Therapy N = 95, Seizure Management N = 28, NG Tube Use in Unconscious Patients N = 35, Appropriate Antibiotic Use N = 147, Complete Adherence to All Guideline N = 147

Complete adherence to severe malaria guidelines was achieved in 3% (n = 4) of patients reviewed (Fig. 3). The greatest strength was in appropriate use of empiric antibiotics, which was achieved in 82% of patients. The most common areas of deficiency were not testing to confirm malaria diagnosis (34%) and inadequate administration of anti-malarial medications (82%).

Of the 50 patients who did not have a positive malaria test documented, 58% (n = 29) had testing ordered that was either not completed or completed but not documented. Fewer than the three recommended doses of artesunate occurred in 22% of eligible patients (n = 32). Additionally, a delay in the administration of the second dose occurred in 67% (n = 78) of patients who received 2 doses, and in the third dose in 77% (n = 71) of patients who received 3 doses. While the recommended time between doses is 12 h, the median interval between dose one and dose two was 15 h (IQR 12–20 h) and the median interval from dose two to dose three was 17 h (IQR 14–25 h).

Other areas of weakness included irregular monitoring of haemoglobin, inadequate administration of transfusions for severe anaemia, inconsistent hypoglycaemia management, lack of oxygen therapy for patients at high risk of hypoxemia (those with respiratory distress, severe anaemia, altered consciousness, or recurrent seizures), and sporadic use of nasogastric tubes for unconscious patients (Fig. 3). In particular, inadequate blood transfusions seem to be clinically meaningful. Among the 40 patients who died, 15 had blood transfusions ordered that they did not receive.

## Discussion

This study sought to determine the current level of adherence to severe malaria treatment guidelines for children at a Ugandan regional hospital and identify opportunities for intervention. Current adherence to severe malaria treatment guidelines was poor at 3%. The most significant areas of deficiency were in malaria testing and artesunate administration. There are resource limitations that contribute to these issues—there are regular stock outs of malaria rapid diagnostic tests and artesunate, the hospital lab is only operational during limited hours, and nurse: patient ratios can be as extreme as 1:100 overnight making proper administration of medications difficult.

Increased supplies of rapid diagnostic tests would allow for more consistent testing prior to antimalarial treatment. Indeed, it has been shown in several studies that the ready availability of testing is a major factor in adherence to the “test and treat” strategy recommended by the WHO [4, 10–14]. Similarly, improved nursing ratios and adequate artesunate supply would allow for more timely administration of medications as well as closer monitoring of patient status. Improvement in these areas is critical to improving outcomes for patients. Delays in malaria treatment lead to progression to more severe forms of disease, decreasing the likelihood of full recovery [15]. Additionally, the inadequate administration or inappropriate use of artesunate may contribute to known evolving drug resistance, which is likely to impact outcomes of future patients [1, 16].

Despite the resource barriers, there are likely potential opportunities for improvement within the existing system. As demonstrated by the research team at Koidu Government Hospital in Sierra Leone, implementing and adhering to a standardized protocol for treatment of severe malaria in a rural hospital with limited resources was effective at decreasing malaria-specific mortality [9]. Through this process they identified leading drivers of mortality and adjusted work flows to ultimately achieve a reduction in malaria-related mortality from 9% to 3.6%. Compliance with the treatment protocol was ensured by twice daily reviews of all relevant patient records by a clinician [9].

Based on this successful model and the particular issues identified in this study, findings were presented to key hospital stakeholders and care providers to develop initial quality improvement interventions. Several recommendations to consider attempting to improve compliance were produced. One is the use of an admission checklist for patients suspected of having severe malaria. This checklist would include confirmation of diagnostic testing and document its results. The checklist also highlights the recommended timing for administration of the three artesunate doses. While rapid diagnostic tests for malaria are not always available, the hospital lab is able to perform microscopy to confirm the diagnosis. To improve the proportion of patients that receive this testing, another change in the admission workflow was proposed—include acquisition of patient blood samples for thick and thin smears by the provider at the time of admission. This will allow for confirmation of malaria diagnosis once the laboratory is open even when the patient is admitted overnight. Lastly, it was proposed to use patient caregivers to assist in timing medications by informing them during ward rounds how many doses are remaining for the child and the times those doses should be administered. This may allow for better caregiver advocacy for drug administration and improved compliance.

Resource limitations also contribute to the other areas of weakness that were identified in this study. Limited laboratory hours and lack of point of care testing decrease the consistency of haemoglobin monitoring despite a high prevalence of severe anaemia and high demand for blood transfusions. Short supply of blood available for transfusions contributes significantly to deaths from severe anaemia. At a public health level, these investigators and hospital stakeholders hope that there can be increased efforts to increase community blood donation and make this vital resource more available. Inadequate supply of glucometer test strips often limits testing for hypoglycaemia. While this will be a difficult barrier to overcome, a different focus for improvement could be on the empiric administration of 10% dextrose for patients with altered consciousness, seizure activity, and poor oral intake. Treating providers acknowledge that oxygen is often provided without being documented, and the same is true for placement of nasogastric tubes in patients with altered consciousness, so documentation of utilization of these items should be improved to better assess algorithm adherence.

This study has several limitations. It is a retrospective review of a convenience sample, and therefore cannot be certain to be representative of all cases at this facility. There can be no assessment of true malaria-related mortality rates as this sample did not include all patients admitted with malaria during this time period. The data obtained were also subject to the quality of documentation in the paper charts, which was incomplete in some cases. It is possible that patients received additional interventions or had test results that were not documented in the reviewed records and, therefore, actual adherence may be higher than what is presented here. The clinical significance of adherence to each component of the guidelines in this study is not known, but given that adherence to standardized treatment protocols has been shown to improve outcomes, this data is still relevant.

Despite the limitations, the data provided here have identified areas of focus for the ongoing malaria treatment quality improvement initiatives in the Paediatrics Wards at Mbale Regional Referral Hospital. The increased awareness that this data has provided for the treatment team allows for increased vigilance in the areas of weakness. Future work will focus on improvements in adherence following implementation of the proposed interventions noted above.

## Conclusions

Current adherence to severe malaria treatment guidelines in children at this Ugandan regional referral hospital is poor, but this study has identified target areas for ongoing malaria treatment quality improvement initiatives. Implementation of an admission checklist for patients suspected of having severe malaria and adjustments to workflow may improve rates of testing for malaria and adherence to anti-malarial administration guidelines.

## Data Availability

The datasets used and/or analysed during the current study are available from the corresponding author on reasonable request.
